# Is there a role for DAZL in human female fertility?

**DOI:** 10.1093/molehr/gaw024

**Published:** 2016-03-16

**Authors:** Roseanne Rosario, Ian R. Adams, Richard A. Anderson

**Affiliations:** 1 MRC Centre for Reproductive Health, Queens Medical Research Institute, University of Edinburgh, Edinburgh EH16 4TJ, UK; 2 MRC Human Genetics Unit, MRC Institute of Genetics and Molecular Medicine, Western General Hospital, Edinburgh EH4 2XU, UK

**Keywords:** DAZL, meiosis, female fertility, oocyte quality, DAZL RNA targets

## Abstract

The RNA binding protein *deleted in azoospermia-like* (Dazl) is a key determinant of germ cell maturation and entry into meiosis in rodents and other animal species. Although the complex phenotype of *Dazl* deficiency in both sexes, with defects at multiple stages of germ cell development and during meiosis, demonstrates its obligate significance in fertility in animal models, its involvement in human fertility is less clear. As an RNA binding protein, identification of the *in vivo* mRNA targets of DAZL is necessary to understand its influence. Thus far, only a small number of Dazl targets have been identified, which typically have pivotal roles in germ cell development and meiotic progression. However, it is likely that there are a number of additional germ cell and meiosis-relevant transcripts whose translation is affected in the absence of Dazl. Efforts to identify these RNA targets have mainly been focused on spermatogenesis, and restricted to mouse. In women, prophase I occurs in fetal life and it is during this period that the ovarian follicle pool is established, thus factors that have a role in determining the quality and quantity of the ovarian reserve may have significant impact on reproductive outcomes later in adult life. Here, we suggest that DAZL may be one such factor, and there is a need for greater understanding of the role of DAZL in human oogenesis and its contribution to lifelong female fertility.

## Introduction

Female reproductive potential is governed by two key factors: oocyte quality and quantity, with the loss of quality underpinning the exponential increase in age-related aneuploidy, and the loss of quantity underlying the age at menopause (Broekmans *et al.*, 2007; Schmidt *et al.*, 2012; Nelson *et al.*, 2013). These factors are increasingly important for women where societal and demographic changes have influenced the age at which they wish to have their families. Indeed, the maternal age at first birth across the Western world has steadily risen, with the number of first-time mothers over the age of 35 years being an all-time high (Schmidt *et al.*, 2012). While an age-related decline in fertility is much less marked in men, defects in spermatogenesis are present in one-third to half of couples who experience infertility (Matzuk and Lamb, 2008; Miyamoto *et al.*, 2015). Thus, reduced gamete quality and quantity is a key issue in both sexes, and although in some cases, techniques such as ICSI and array comparative genomic hybridization to identify euploid embryos can provide ways of circumventing the problem, they do not address the underlying issues.

The establishment of the ovarian reserve occurs during fetal life and begins with the migration of primordial germ cells into the gonadal ridge during early gestation (reviewed in Findlay *et al.*, 2015), although post-natal oogenesis is also debated (Grieve *et al.*, 2015). Here the germ cells undergo mitotic divisions with incomplete cytokinesis, producing a surplus of interconnected oogonia within germ cell ‘nests’ (Gondos *et al.*, 1971; Pepling and Spradling, 1998). Ceasing proliferation, the oogonia enlarge and develop into primary oocytes, which commence meiosis I and progress through leptotene, zygotene and pachytene before arresting at diplotene of prophase I. The ‘nest’ structure undergoes breakdown, in which most oocytes are lost through apoptosis, while the remainder are enclosed by a layer of pregranulosa cells, thus forming primordial follicles from 17 weeks gestation. These oocytes remain quiescent in a state of meiotic arrest until, should they escape the atresia that befalls >99% of all follicles, they are reactivated at the time of ovulation: this will not be months or years, but decades later. Greater understanding of how oocytes maintain long-term meiotic arrest with chromosomal fidelity is critical to our knowledge of lifelong reproductive potential in women, and may provide an opportunity for both diagnosis and therapy.

## DAZL (deleted in azoospermia-like)

**Figure 1:**
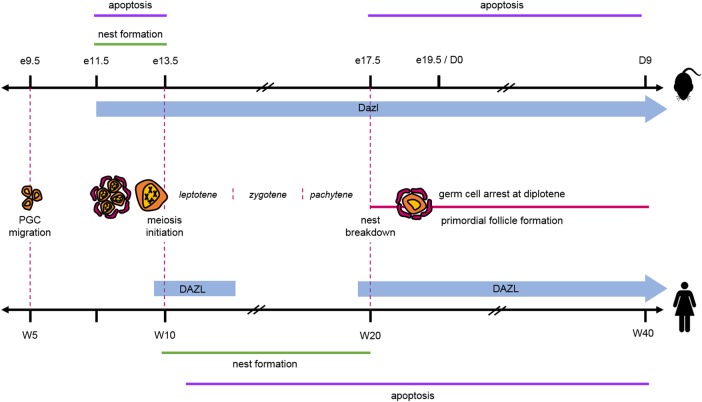
DAZL (deleted in azoospermia-like) expression during formation of the ovarian reserve. DAZL is first expressed in germ cells prior to meiosis in human and mouse. While this continues in mouse until after follicle formation, in humans, DAZL is switched off briefly before being re-expressed in oocytes of primordial follicles. E, embryonic day; D, post-natal day; PGC, primordial germ cell; W, weeks gestation.

*DAZL* and homologue members *DAZ* and *BOLL* (previously known as *BOULE*) belong to the DAZ family of genes encoding RNA binding proteins, and have essential roles in gametogenesis. It is likely that *BOLL* is the ancestral gene, first identified in Drosophila (where it is known as *bol*), and *DAZL* is considered to have arisen from *BOLL* through a gene duplication event (Castrillon *et al.*, 1993). While *BOLL* appears to be strongly evolutionarily conserved, *DAZL* is only found in vertebrates and *DAZ* is limited to just humans and old world monkeys (Reijo *et al.*, 1995). *DAZL* is a single-copy autosomal gene and encodes a protein that is specifically expressed in germ cells at all stages of oogenesis. In addition, DAZL expression has been reported in human and mouse granulosa cells, human theca interna cells and in granulosa-luteal cells of the human corpus luteum; however, these remain controversial (Ruggiu *et al.*, 1997; Dorfman *et al.*, 1999; Nishi *et al.*, 1999; Pan *et al.*, 2002). In mouse fetal gonads, *Dazl* transcripts are first detected from embryonic day (e) 11.5 in post-migratory germ cells (Fig. 1), and this activation of *Dazl* appears to be a consequence of the genome-wide loss of DNA methylation that occurs in germ cells at this stage (Hackett *et al.*, 2012). *Dazl* expression dramatically increases at e13.5 in females, coinciding with the onset of meiosis (Seligman and Page, 1998), and by e17.5 transcript expression in the ovary steadily decreases as the number of oocytes is depleted. In the human fetal ovary, a marked increase in DAZL mRNA and protein is observed between 9 and 14 weeks gestation (Ruggiu and Cooke, 2000; Anderson *et al.*, 2007; He *et al.*, 2013), that immediately precedes and is coincident with the onset of meiosis. At this time, DAZL undergoes a dynamic redistribution from the germ cell nucleus (where it may have a role in RNA processing and storage) to the germ cell cytoplasm where it is expected to function (Reynolds and Cooke, 2005; Anderson *et al.*, 2007). Expression of DAZL is also found in oocytes of newly formed primordial follicles (He *et al.*, 2013) and in mouse, Dazl expression been demonstrated to persist through later stages of oocyte maturation, including through to zygote formation, where Dazl-dependent translation is necessary for spindle assembly, the metaphase I–II transition and early embryo development (Chen *et al.*, 2011).

The phenotype of Dazl deficiency has been investigated in detail in the mouse. The deletion of *Dazl* results in a loss of germ cells in both male and female gonads, with evidence of increased apoptosis, reduced expression of germ cell markers and aberrant chromatin structure (Ruggiu *et al.*, 1997; Schrans-Stassen *et al.*, 2001; Lin and Page, 2005). The loss of *Dazl* leads to a reduction in the number of post-migratory primordial germ cells, defects in sexual differentiation of the germ cells, a failure to erase and re-establish genomic imprints, and defects in meiotic progression (Haston *et al.*, 2009; Gill *et al.*, 2011). The defect in meiosis has been shown by immunohistological analysis to be a failure to progress beyond leptotene of meiotic prophase I. Although axial elements are formed [visualized using an antibody against synaptonemal complex protein 3 (Sycp3)], complete synaptonemal complexes are not, giving a precise point beyond which meiosis cannot progress without *Dazl* (Saunders *et al.*, 2003). Transplantation of ROSA/*LacZ*-labelled spermatogonia into the testes of *Dazl^−/−^* mice confirms that the somatic tissues are capable of supporting gamete development, indicating that it is only germ cell function that is compromised in these animals (Rilianawati *et al.*, 2003).

Many RNA binding proteins are recognized as having roles in translational control during gametogenesis. During oogenesis, growing oocytes accumulate significant quantities of mRNAs, but transcription ceases during the final stages of maturation and subsequent protein production is dependent upon a well-orchestrated programme of dynamic modulation of mRNA poly(A) tail length, recruitment to polysomes and translational activation (Seydoux and Braun, 2006; Radford *et al.*, 2008). Indeed, DAZL is associated with actively translating polysomes (Tsui *et al.*, 2000b; Maegawa *et al.*, 2002) and sucrose gradient analysis of translation intermediates from a 3′ untranslated region (UTR) tethering reporter assay in *Xenopus laevis* oocytes revealed that mDazl acts to specifically stimulate translation through regulation of the initiation stage (Collier *et al.*, 2005). DAZL contains two functional domains: a highly conserved RNA recognition motif (RRM) and a single DAZ domain unique to DAZ family proteins. Investigation into the binding specificity of Dazl using RNA homopolymers demonstrated that the RRM of *Xenopus* Dazl preferentially binds poly(U) and poly(G) RNA *in vitro* (Houston *et al.*, 1998), and this was also the case for human DAZL (Tsui *et al.*, 2000b). An improvement to the description of this binding element was achieved using SELEX, a powerful RNA ligand enrichment-based strategy, in addition to a three-hybrid screen, to show that mDazl binds oligo(U) stretches interspersed by G or C nucleotides (Venables *et al.*, 2001). When the G or C nucleotides were replaced by A, only a weak interaction was observed between Dazl and the binding site, and replacement with a U abolished this interaction completely. Resolution of the RRM crystal structure of mDazl with and without 3′UTR target sequences has revealed the involvement of an extended, kinked pair of β-strands in the recognition of a GUU triplet in the 3′UTR of Dazl targets (Jenkins *et al.*, 2011). The nucleotide at position 4 of this refined Dazl consensus sequence varies between complexes (U or C), and mutation of bases within the GUU triplet reduces the affinity of binding. Searching for DAZL targets based on this binding site alone is likely to return many false positives given that such short sequences may occur relatively frequently in many mRNAs. Therefore, the presence of multiple DAZL-binding elements, which can enhance translational stimulation (Collier *et al.*, 2005; Fox *et al.*, 2005), partner proteins such as DAZAP1 or PUM2 (Tsui *et al.*, 2000a; Moore *et al.*, 2003), which may modulate DAZL stimulation, and appropriately timed germ cell expression should all be taken into consideration when attempting to uncover DAZL targets.

## DAZL in fertility: action through its mRNA targets

Efforts to identify *in vivo* mRNA targets of mammalian DAZL have mainly been focused on mouse. The results of these inquiries further attest to Dazl's role in fertility as many identified targets are key players in germ cell development and gametogenesis, particularly during meiosis. DAZL could potentially have distinct mRNA targets at different stages of germ cell development. The first screen for Dazl targets, carried out as part of an investigation addressing the absence of DAZ proteins and spermatogenic defects, identified *Tpx-1,* a testicular cell adhesion protein (Jiao *et al.*, 2002). The significance of *Tpx-1* breakpoints in male fertility, together with its temporal expression pattern, suggests this gene is essential for the progression of spermatogenesis, and Dazl has a key role in regulating it*. Tpx-1* is not expressed in the female, but a study in mouse oocytes has identified *Tpx-2* as a Dazl target (Chen *et al.*, 2011). TPX2 activates a regulator of centrosome and spindle pole assembly, and itself participates in spindle pole organization (Gruss *et al.*, 2001; Bayliss *et al.*, 2003). Morpholino down-regulation of *Tpx-2* in oocytes causes spindle assembly disruption and chromosome condensation failure; phenotypic changes similar to those observed post-Dazl knockdown in oocytes (Brunet *et al.*, 2008), suggesting Tpx2 protein is responsible for these defects and may be a key target for Dazl in post-natal mouse oocytes. These studies used *in vivo* matured germinal vesicle, metaphase I and metaphase II mouse oocytes and also confirm *Tex19.1,* which has a role in both male and female fertility in mice (Ollinger *et al.*, 2008), as a bona fide Dazl target.

*Tex19.1* had previously been reported as a potential Dazl target during spermatogenesis along with *Sycp3* and *Vasa* (formally known as *DDX4*, or *Mvh* in mouse) (Reynolds *et al.*, 2005). These genes were identified as potential targets for translational regulation by Dazl using a combination of immunoprecipitation and microarray in male mouse germ cells from wildtype and Dazl^−/−^ animals, making these studies the first attempt to isolate endogenous Dazl–RNA complexes rather than exploiting interactions with recombinant proteins or RNA libraries (Reynolds *et al.*, 2005, 2007). Sequence analysis showed that *Sycp3* and *Vasa* transcripts contain Dazl binding elements, and RNA binding and luciferase assays were used to demonstrate that Dazl stimulates transcript translation via the 3′UTR; in the case of *Sycp3*, this response was diminished upon mutation of the Dazl binding sites. VASA is an ATP-dependent RNA helicase of the DEAD-box family (Raz, 2000), and is widely utilized as a germ cell marker, while SYCP3 is a major component of the lateral elements of the synaptonemal complex in meiotic germ cells (Schramm *et al.*, 2011). Defects in translation of *Vasa*, *Sycp3* and *Tex19.1* could all contribute to the defects in meiotic progression reported in *Dazl*^−/−^ spermatocytes (Saunders *et al.*, 2003), as *Mvh*^−/−^, *Sycp3*^−/−^ and *Tex19.1*^−/−^ spermatocytes all have defects in progression from leptotene through to pachytene stages of meiosis I (Tanaka *et al.*, 2000; Ollinger *et al.*, 2008). However, some aspects of the male *Dazl*^−/−^ phenotype, such as the defects in spermatogonial differentiation (Schrans-Stassen *et al.*, 2001), the reduction in post-migratory germ cell number (Haston *et al.*, 2009), extensive germ cell apoptosis during male fetal development (Lin and Page, 2005) and the failure of fetal oocytes to progress through meiotic prophase (Ruggiu *et al.*, 1997; Saunders *et al.*, 2003), are not reported in any of the *Mvh^−/−^*, *Sycp3*^−/−^ or *Tex19*.*1*^−/−^ mice, suggesting that additional as yet unidentified Dazl targets are likely contributing to the complex *Dazl^−/−^* phenotype.

DAZL may also impact fertility through its mRNA targets with known functions in the regulation of pluripotency, differentiation and apoptosis. Through an association with *Tet1*, Dazl has an essential role in mediating the hydroxylation of methylated cytosine residues in mouse embryonic stem cells that are actively reprogramming to a pluripotent ground state (Welling *et al.*, 2015). In primordial germ cell-like cells derived from mice embryonic stem cells, DAZL was demonstrated to block the translation of core pluripotency factors *Sox2* and *Sall4,* which are essential for stem cell self-renewal, as well as of *Suz12*, which is required for the differentiation of pluripotent cells (Chen *et al.*, 2014). In addition, Dazl inhibited the translation of proapoptotic caspases, which is consistent with the *Dazl*^−/−^ phenotype, in which embryos have a drastic reduction in germ cell number due to increased apoptosis (Lin and Page, 2005; Chen *et al.*, 2014). Although it is mostly recognized to enhance translation, this is one of few instances where a repressive function has been reported for Dazl. It is possible that the observed translational repression by Dazl in these investigations is an indirect effect, as some of Dazl's partner proteins, for example, Pumilio-2 (Moore *et al.*, 2003; Fox *et al.*, 2005), are known to bind RNA and function as translational repressors. Nevertheless, it appears that DAZL has a more complex role in regulating its downstream target transcripts than was previously believed, although these data also highlight the need for an understanding of which effects are through direct binding of the mRNA target and which are indirect.

## Is DAZL relevant to human female fertility?

In humans, direct demonstration of a causative role is not possible, but observation of the association of DAZL with reproductive outcomes provides some evidence for the involvement of this gene in human fertility. However, given the protracted duration of oogenesis and the inaccessibility of the female gamete, the majority of findings pertaining to DAZL function have been restricted to the testis in both experimental animals and humans. In males, it is estimated that 10–15% of cases of azoopermia and oligozoospermia result from deletions within the Y chromosome, specifically the region which contains the *DAZ* gene cluster (Reijo *et al.*, 1995), but *DAZL* is considered to be a prime candidate implicated in roughly 60% of idiopathic male infertility, given its high similarity to *DAZ,* its germ-cell specific expression and the suspected involvement of autosomal recessive mutations in these men (Lilford *et al.*, 1994; Visser and Repping, 2010). Altered methylation of the *DAZL* promoter has been associated with male infertility (Navarro-Costa *et al.*, 2010; Li *et al.*, 2013). Furthermore, polymorphisms in both the promoter and coding sequence of *DAZL* have been correlated with total sperm count, sperm motility, age at menopause and primary ovarian insufficiency (POI), also known as premature ovarian failure, in infertile men and women, respectively (Teng *et al.*, 2002; Tung *et al.*, 2006a), although other studies have been unable to find such associations (Bartoloni *et al.*, 2004; Zerbetto *et al.*, 2008). Interestingly, many of the allelic variants studied exhibited opposing effects in men and women, which may be indicative of the different genetic requirements of male and female germ cells (Tung *et al.*, 2006b). This sex-dependent effect was also observed in knockout mice, where the *Dazl^−/−^* phenotype could be partly rescued by a human DAZL transgene in male mice, but not in females (Vogel *et al.*, 2002). Ultimately, these observations suggest that hypomorphic polymorphisms and single-nucleotide polymorphisms that quantitatively reduce DAZL expression or function in human germ cells could potentially have consequences for fertility.

Thus far, the study by Tung *et al.* (2006a, b) has been the only report of DAZL's relevance to human female fertility and while Tung *et al.* suggest that DAZL might affect oocyte quantity in women (through influencing age at POI or menopause), there has been no examination as to whether the quality of oocytes is affected too. Given that DAZL was shown to influence total sperm count and sperm motility, quantity and quality parameters respectively, it seems possible that the same should occur in women; a key significant difference being the developmental period at which the effect happens. The generation of sperm via spermatogenesis, and thus meiosis, is a continuous process beginning at the onset of puberty and continuing throughout male adult life. However, in females, the process of oogenesis is initiated in the fetus, with the formation of stored oocytes that are used periodically over a defined reproductive lifespan. It is not clear if the genetic associations between DAZL polymorphisms and age at menopause and POI reflect a role for DAZL in fetal oocyte development or maintenance of oocytes during their prolonged post-natal arrest, but these associations are consistent with the fetal oocyte death reported in Dazl null mice (Ruggiu *et al.*, 1997; Saunders *et al.*, 2003), and provide a link between DAZL function during fetal/early post-natal life and reproductive success later in adult life.

The increase in the incidence of infertility and miscarriage from the mid-fourth decade of female life onwards (Schmidt *et al.*, 2012) is in great part attributed to segregation errors in meiosis I in the female (Hassold and Hunt, 2001). This is likely because oocytes that are ovulated in late reproductive life differ from their earlier counterparts in the duration of prophase I arrest. The requirement for DAZL during meiotic progression in fetal mouse oocytes suggests that less severe hypomorphic mutations and/or polymorphisms in DAZL in humans could perturb meiotic progression sufficiently to impair oocyte quality. Accurate chromosome segregation in adult oocytes depends on successful establishment of meiotic crossovers between paired and synapsed homologous chromosomes during fetal development, and the subsequent maintenance of those crossovers post-natally (Herbert *et al.*, 2015; MacLennan *et al.*, 2015). Defects in meiotic progression during fetal oocyte development, such as those reported in female mice carrying mutations in the Dazl-target Sycp3, can result in aneuploidy arising during the meiotic chromosome segregations and reduced oocyte quality in adulthood, although this is not age-related (Yuan *et al.*, 2002). However, analysis of chromosomes in oocytes of naturally aged mice demonstrated that premature bivalent separation into univalents is the primary defect responsible for age-related aneuploidy (Sakakibara *et al.*, 2015). This was also found in human oocytes, where there was an increase in sister kinetochore separation, rotated bivalents and merotelic attachments, suggesting in fact there are multiple age-related changes in chromosome architecture with advanced maternal age (Zielinska *et al.*, 2015). Therefore, the potential role of DAZL, and DAZL-targets, in meiotic progression in human fetal oocytes may be able to impact on human female fertility in adulthood through influencing oocyte quality. Experiments in mice suggest that Dazl also has functions in fully grown oocytes in adult life where it is essential for the resumption of meiosis I in germinal vesicle oocytes and the transition to meiosis II, as in its absence, meiotic microtubules become disorganized causing meiotic spindle failure; these oocytes cannot be fertilized (Chen *et al.*, 2011). Consequently, factors acting during oogenesis at the time of meiosis initiation, arrest and completion can potentially have fundamental roles in determining oocyte quality as well as quantity, thereby influencing both components of reproductive lifespan: a women's ability to conceive later in life as well as her age at menopause. We suggest that *DAZL* may be one such factor, and for this reason, highlight the need for specifically female-oriented studies to investigate this notion further.

## Experimental strategies to investigate the role of DAZL in women

The identification of DAZL mRNA targets illustrates the growing spectrum of processes in gametogenesis that this gene is proposed to regulate. However, the only work in humans to date, where overexpression of DAZL elevated endogenous VASA and SYCP3 levels (Kee *et al.*, 2009), has been in embryonic stem cells rather than germ cells. The study of DAZL in women is complicated by scarce access to female gametes. However, it is possible to obtain fetal ovarian tissue after elective termination of pregnancy, while mature adult oocytes may be accessed in the course of IVF or ICSI cycles, and advances in follicle culture (Xiao *et al.*, 2015) may allow access across a range of developmental stages. Further understanding of the role of DAZL in oogenesis will require knowledge of its target mRNAs at different stages throughout development. The most ideal way to achieve this would be to adopt an RNA immunoprecipitation approach similar to that of Reynolds and Cooke (2005) in order to isolate RNAs bound to endogenous DAZL. These RNAs can then be identified using microarray or high-throughput RNA sequencing; advances in these technologies now make this possible with limited amounts of starting material (Kanter and Kalisky, 2015). However, limitations include the sensitivity of the assay, as well as the strength of the DAZL–RNA interaction. Cell lysis can also lead to associations between molecules that are not representative of interactions that occur *in vivo*, and for this reason, newly identified targets should be investigated *in vitro* using RNA binding assays and polysome profiling to confirm DAZL's ability to regulate target RNA translation. Lastly, gene manipulation of DAZL using RNA interference in cultured human fetal ovaries or mature oocytes provides a powerful strategy to explore the functional significance of novel RNA targets during meiosis, fertilization and early embryo formation. This approach has been used to study the roles of growth differentiation factor 9 and proliferating cell nuclear antigen in newborn hamster ovaries (Wang and Roy, 2006; Xu *et al.*, 2011), and of DMRT-Like Family A2 (DMRTA2) in the human (Poulain *et al.*, 2014). Alternatively, silencing of DAZL or RNA target gene expression in mature oocytes can be achieved through the injection of specific antisense morpholino oligonucleotides (Chen *et al.*, 2011).

## Concluding remarks

The RNA binding protein DAZL has an essential role in translational control during gametogenesis, and its absence or altered function is associated with infertility in several organisms, including humans. Although many targets of DAZL have been investigated *in vitro* and *in vivo* in a number of different species, many, or indeed probably most of its mRNA targets have yet to be identified, especially in women. As a key factor in the initiation of meiosis, DAZL target mRNAs involved with this are of particular significance, given the critical issues in meiosis specific to female gamete formation. Therefore, identification of mRNAs that depend on DAZL for regulation of their translation will increase our understanding of the fundamental processes that underpin oocyte quality and quantity, which in turn determine a woman's reproductive potential and reproductive lifespan.

## Authors' roles

R.R. wrote the manuscript. I.R.A. and R.A.A. edited the manuscript.

## Funding

The authors' work in this field is supported by grants from the Medical Research Council (G1100357 to R.A.A. and an intramural program grant to I.R.A.). Funding to pay the Open Access publication charges for this article was provided by the Medical Research Council.

## Conflict of interest

None declared.
